# Endocrine and Metabolic Manifestations of COVID-19 Patients Admitted to an Intensive Care Unit

**DOI:** 10.7759/cureus.24702

**Published:** 2022-05-03

**Authors:** Saurabh Arora, Akashdeep Singh, Vipin Kumar, Bishav Mohan, Rajesh Mahajan, Navdeep Singh, Parminder Singh, Naveen Mittal, Suman Sethi, Sarit Sharma, Sanjay Kalra, Nitin Kapoor, Saloni Goyal

**Affiliations:** 1 Department of Endocrinology, Dayanand Medical College and Hospital, Ludhiana, IND; 2 Department of Pulmonary Medicine, Dayanand Medical College and Hospital, Ludhiana, IND; 3 Department of Internal Medicine, Dayanand Medical College and Hospital, Ludhiana, IND; 4 Department of Cardiology, Hero Dayanand Medical College and Hospital (DMC) Heart Centre, Ludhiana, IND; 5 Department of Internal Medicine, Wilkes-Barre General Hospital, Wilkes-Barre, USA; 6 Department of Social and Preventive Medicine, Dayanand Medical College and Hospital, Ludhiana, IND; 7 Department of Endocrinology, Bharti Hospital and Bharti Research Institute of Diabetes and Endocrinology (BRIDE), Karnal, IND; 8 Department of Endocrinology, Christian Medical College and Hospital, Vellore, IND; 9 Department of Pathology, Dayanand Medical College and Hospital, Ludhiana, IND

**Keywords:** hypocalcemia, diabetic ketoacidosis, thyrotoxicosis, rhino-orbital mucormycosis, pulmonary mucormycosis

## Abstract

Context: The effects of coronavirus disease 2019 (COVID-19) on the endocrine system remain uncertain.

Objective: Our study aimed to explore the possible effects of COVID-19 on endocrine organs and to determine the impact of glycemic status, 25-hydroxyvitamin D levels, calcium levels, and thyroid dysfunction on the final outcome of patients with COVID-19.

Design and methods: This single-center, retrospective study evaluated endocrine function abnormalities in 102 patients hospitalized with COVID-19 in the intensive care unit (ICU).

Results: Of 102 patients admitted to ICU, 42 (41.2%) succumbed to illness. The most frequently observed abnormality in thyroid function tests was low free triiodothyronine (FT3) levels (56%). A thyroid profile indicating thyrotoxicosis was detected in five (4.9%) patients, and overt hypothyroidism was identified in two (1.9%) patients. New-onset diabetes was detected in five (4.9%) patients whereas diabetic ketoacidosis at presentation was found in six (5.9%) cases. Rhino-orbital mucormycosis was detected in one patient with diabetes during treatment of COVID-19 while three (2.9%) patients were diagnosed with pulmonary mucormycosis after recovery from COVID-19. Hypocalcemia was observed in 52 (51 %) patients. Out of 42 patients who died, 32 patients had low FT3, 26 patients had high glycated haemoglobin (HbA1c), and 33 patients had low 25-hydroxyvitamin D. Multivariate analysis demonstrated that low concentration of 25-hydroxyvitamin D, low FT3 and higher HbA1c levels were significantly associated with increased mortality.

Conclusion: New-onset thyrotoxicosis in COVID-19 patients is mostly due to subacute thyroiditis. Hypocalcemia is also frequently encountered in patients with moderate disease and those with critical COVID-19. A high index of suspicion is required to timely diagnose mucormycosis in COVID-19 patients with diabetes.

## Introduction

The COVID-19 pandemic has spread at an alarming pace, affecting more than 500 million people globally and disrupting the everyday life of all people. Different studies around the world have shown that COVID-19 can cause moderate to severe pneumonia in approximately 15% of infected individuals and lead to life-threatening consequences if managed inappropriately. Myocarditis, acute respiratory distress syndrome, and acute kidney injury are the well-established life-threatening complications of COVID-19. On the other hand, the endocrinological manifestations of COVID-19 in India have not been studied in detail.

Severe acute respiratory syndrome coronavirus 2 (SARS-CoV-2) utilizes angiotensin-converting enzyme 2 (ACE2) as a receptor to enter host pneumocytes followed by viremia [1]. Both animals and humans show varying expression of ACE 2 in endocrine organs i.e., hypothalamus, pituitary gland, thyroid gland, gonads, and pancreatic islets [2]. Expressing this receptor allows the virus to enter endocrine cells and disrupt their function. Patients with underlying diabetes mellitus are more predisposed to develop severe or critical COVID-19 disease compared with patients without diabetes [3]. On the other hand, new-onset diabetes mellitus with diabetic ketoacidosis or glycemic control deterioration have been reported in patients with preexisting diabetes [4]. Lui et al. have reported thyroid dysfunction in approximately 15% of patients with mild to moderate COVID-19 [5]. Given the scarcity of evidence on COVID-19 endocrine manifestations, our study aimed to explore the possible effects of COVID-19 on the endocrine organs.

## Materials and methods

Medical records of 102 patients with laboratory confirmed COVID-19 that were admitted to an intensive care unit (ICU) of Dayanand Medical College and Hospital, Ludhiana, Punjab, between September 1, 2020, and December 31, 2020, were retrospectively reviewed. All patients included in this study tested positive for SARS-CoV-2 by quantitative reverse-transcriptase polymerase chain reaction based on samples acquired from their nasopharynx. All patients were divided into three clinical categories: moderate, severe, and critical depending on their clinical presentation and according to the World Health Organization severity classification [6]. Patients with mild COVID-19 were admitted to medical wards and thus not included in this study. Data on comorbidities, new-onset diabetes, presentation with diabetic ketoacidosis, HbA1C level, thyroid function tests, 25-hydroxyvitamin D level, and serum calcium, magnesium, and phosphorous levels were extracted from the medical records. Based on the American Diabetes Association Consensus Statement on Inpatient Glycemic Control, hyperglycemia in hospitalized patients was defined as any blood glucose value ≥ 140 mg/dL (≥ 7.8 mmol/L) [7]. Thus, patients were categorized as normoglycemic with all blood glucose recordings <140 mg/dL or hyperglycemic with any blood glucose level of ≥ 140 mg/dL during the first 24 hours of admission. Cases were excluded from the study if the above-mentioned parameters were not measured within the first 72 hours of admission, or the patients had a history of thyroid, pituitary, or adrenal disease in the past and/or were taking thyroid hormones, antithyroid medications, or corticosteroids before admission.

Statistical analysis

Statistical analysis was performed using SPSS Statistics for Windows, Version 20.0 (IBM Corp., Armonk, NY). The Student's t-test was used to determine the difference between the means of two independent groups while the Chi square test was used to determine the difference between two proportions. Multivariate logistic regression analysis was used to identify the association of selected variables with mortality. P < 0.05 was considered to indicate statistical significance. Since there was no control group in this observational study, the odds ratio for in-hospital mortality was calculated by comparing subjects who were having risk factors with those who were not having risk factors. 

## Results

Baseline characteristics

Of 102 patients, 60 (58.8%) patients had a severe disease while 24 (23.5%) had a critical disease. The remaining 18 (17.7%) patients had a moderate disease. Baseline characteristics of the patients are shown in Table 1. In our study, 41.2% of patients in an ICU succumbed to the COVID-19, while 58.8% of patients were shifted to medical wards after clinical condition stabilization. Diabetes mellitus was the most common comorbidity (58.8%), followed by hypertension (39.2%) and cardiovascular disease (19.6%).

**Table 1:** Baseline clinical characteristics of 102 patients IR, interquartile range; SD, standard deviation; HbA1c, glycated haemoglobin; T3, triiodothyronine; T4, thyroxine; TSH, thyroid-stimulating hormone

Variable	N (%)
Age	55.5 ± 14.8
Male: female	3.25:1
Severity of disease	
Moderate COVID-19	18 (17.7)
Severe COVID-19	60 (58.8)
Critical COVID-19	24 (23.5)
Diabetes	60 (58.8)
Hypertension	40 (39.2)
Cardiovascular disease	20 (19.6)
Chronic kidney disease	12 (11.8)
Chronic liver disease	10 (9.8)
Free T3 ( pmol/l, median)	2.98 ( IR 2.17 – 3.46)
Free T4 ( pmol/l, median)	14.67 ( IR 13.62 – 16.46)
TSH (µIU/ml, median)	1.10 ( IR 0.53 – 1.62)
HbA1c (%, mean±SD)	7.82 ± 2.0
25-hydroxyvitamin D3 (ng/ml, median)	20.54 ( IR 10.68 – 32.84)
Corrected calcium (mg/dl, mean±SD)	8.34 ± 0.66
C- reactive protein (mg/l, median)	128.08 ( IR 42.28 – 200.25)

COVID-19 and thyroid dysfunction

The most frequently observed pattern of thyroid function test in our study was non-thyroidal illness syndrome (58.8%) followed by euthyroid state (35.3%). A thyroid profile indicating thyrotoxicosis was detected in five (4.9%) patients, and overt hypothyroidism was identified in two (1.9%) patients for the first time. All five patients with thyrotoxicosis were subsequently diagnosed with subacute thyroiditis based on either absent uptake on technetium thyroid scan or negative thyroid-stimulating hormone (TSH) receptor antibody test. Of these five patients, one had moderate COVID-19, and the remaining four had severe COVID-19. Two patients with biochemically confirmed thyrotoxicosis died due to hypoxia worsening, while three patients recovered from COVID-19. Technetium 99 m pertechnate thyroid scintigraphy was performed in COVID-19 survivors after the patients were discharged from the isolation ward. All patients demonstrated reduced to absent uptake on thyroid scintigraphy.

Logistic regression analysis demonstrated that low FT3 levels showed a significant association with increased severity and mortality (p = 0.002) whereas free thyroxine (FT4) and TSH did not affect the outcomes. The presence of subacute thyroiditis or overt hypothyroidism did not impact the outcome as well (p > 0.05).

COVID-19 and diabetes mellitus

Of 60 (58.8%) patients with diabetes mellitus, 51 (50%) patients were previously diagnosed with diabetes while nine (8.8%) patients were diagnosed with diabetes mellitus only after admission. Among patients with newly detected diabetes, five (55.5%) patients had HbA1c of less than 6.5% at admission and were considered to have new-onset diabetes mellitus. Diabetic ketoacidosis was present at admission in two (1.9%) newly diagnosed patients, whereas one (0.9%) patient presented with a hyperosmolar hyperglycemic state as initial diabetes manifestation. Out of 51 patients with a previous history of diabetes, three (2.9%) patients with type 2 diabetes and one (0.9%) patient with type 1 diabetes presented with diabetic ketoacidosis. Hyperglycemia at presentation was detected in 64 (62.7%) patients, and mortality was significantly higher in the hyperglycemia group as compared to the normoglycemia group (68.8% vs. 42.1%, p = 0.014). Patients who survived had significantly lower HbA1c levels as compared to those who died (7.5% vs. 8.9%, p = 0.002).

Mucormycosis was diagnosed in four (3.9%) patients who had severe COVID-19 disease, uncontrolled diabetes, and had received immunosuppressive therapy. Rhino-orbital mucormycosis was detected in one patient with diabetes after 28 days of admission to an ICU while three patients presented with pulmonary mucormycosis during follow-up. All patients had underlying type 2 diabetes, and none of them had diabetic ketoacidosis at presentation. The patient with rhino-orbito-cerebral mucormycosis also had chronic kidney disease stage 5 besides type 2 diabetes and had received 800 mg tocilizumab three weeks before the diagnosis of mucormycosis. Mucormycosis was diagnosed based on direct microscopy of a specimen from nasal sinus mucosa, and the patient was prescribed antifungals but succumbed to mucormycosis on day 32. Pulmonary mucormycosis was diagnosed based on a report of fine-needle aspiration cytology from pulmonary tissue in all three patients after recovery from the acute COVID-19 phase. All patients had received pulse steroid therapy (methylprednisolone 500 mg/day for three days) for cytokine storm syndrome associated with COVID-19 during the previous hospitalization, and the mean duration of mucormycosis diagnosis from discharge was 25.6 days. Patients with pulmonary mucormycosis were treated with liposomal amphotericin B, however, progressive dissemination could not be stopped, and all patients died. The mean HbA1c of all COVID-19 patients who were infected with mucormycosis was 8.9 %.

COVID-19 and hypocalcemia

The mean corrected total calcium level in patients with COVID-19 was 8.34 ± 0.66 mg/dl. Almost one-half (n= 52) of patients in the COVID-19 group had hypocalcemia (total corrected calcium of less than 8.8 mg/dl ), and 46% (n = 47) of patients had vitamin D deficiency (25- hydroxyvitamin D level of less than 20 ng/ml). Hypocalcemia was observed in 44.4 %, 48.3%, and 62.5% of patients with moderate, severe, and critical COVID-19, respectively. Hypocalcemia prevalence increased with disease severity but the difference was statistically insignificant (p = 0.41). Of 102 patients with COVID-19, eight (7.8%) patients had hypocalcemia associated with hyperphosphatemia and renal failure was identified in four of these patients. The remaining four patients with biochemically confirmed hypocalcemia associated with hyperphosphatemia showed normal renal function and all four patients had severe-to-critical COVID-19. The mean parathyroid hormone (PTH) and 25-hydroxyvitamin D concentrations in these patients were 51.5 pg/ml and 21.5 ng/ml, respectively. Multivariate logistic regression analysis demonstrated that lower levels of 25-hydroxyvitamin D were associated with increased mortality (p = 0.001 ), whereas low calcium concentration did not significantly affect the outcome (p = 0.98). The results of multivariate regression analysis for the prediction of in-hospital mortality are provided in Table 2.

**Table 2:** Results of multivariate logistic regression analysis to predict in-hospital mortality in COVID-19 patients HbA1C, glycated hemoglobin; T3, triiodothyronine; T4, thyroxine; TSH, thyroid-stimulating hormone

Variable	P-value	Odds ratio	95% Confidence interval	
High HbA1C	0.002	1.724	1.229	2.417
Low free T3	0.002	5.135	1.891	14.94
Low free T4	0.949	0.994	0.820	1.205
TSH	0.136	0.814	0.620	1.067
Low 25-hydroxyvitamin D	0.001	9.627	3.374	27.42
Corrected calcium	0.989	0.994	0.431	2.296
Phosphorous	0.108	1.295	0.945	1.774

## Discussion

This study highlights various endocrine abnormalities and their impact on mortality among hospitalized patients with COVID-19. Abnormal thyroid function tests were the most common finding, followed by hypocalcemia. The most frequently observed abnormal thyroid function pattern was low FT3 level with normal FT4 and TSH levels indicating non-thyroidal illness syndrome. This study showed that reduced FT3 levels were significantly associated with severe disease and increased all-cause mortality. Gao et al. [8] analyzed the data of 100 patients with COVID- 19 and concluded that low FT3 concentration independently predicted higher mortality. Increased levels of proinflammatory cytokines, such as interleukin (IL)-6 and tumour necrosis factor (TNF )-alpha, were suggested to play a role in non-thyroidal illness syndrome pathogenesis [9]. In our study, IL-6 levels were not routinely measured in all patients, therefore we cannot speculate on the relationship between low FT3 and IL-6 levels. Our study did not report any significant association of low TSH and low FT4 with outcomes in COVID-19 patients admitted to an ICU. A retrospective study performed in Italy showed that in-hospital mortality among patients with COVID-19 was significantly lower in those with normal TSH compared to patients with either high or low TSH levels [10].

In the current study, thyrotoxicosis was reported in 4.9% of patients and the possibility of destructive thyroiditis was considered in all patients in the view of thyrotropin receptor antibody (TRAB) negativity and thyroiditis self-resolution during follow up in all three patients who survived COVID-19. The possibility of destructive thyroiditis was further supported by decreased-to-absent uptake on a technetium thyroid scan. All patients were managed with beta-blockers, glucocorticoids, and cholestyramine. The literature demonstrates the high expression of ACE 2 in the thyroid gland [11], which is the protein utilized by SARS-CoV-2 to enter human cells. A possibility of direct SARS-CoV-2 effect on thyroid tissue could not be ruled out at this point requiring further research to clarify the mechanism responsible for thyroiditis in COVID-19 patients. Another study from Italy reported a higher prevalence (20.2%) of thyrotoxicosis in COVID-19 patients [10], whereas Khoo et al. [12] did not report any case of thyrotoxicosis in a study on 334 COVID-19 patients. Current evidence suggests that the clinical manifestations of thyroid involvement in patients infected with SARS-CoV-2 are not consistent worldwide.

Our study evaluated the relationship between hyperglycemia and outcomes in COVID-19 patients admitted to an ICU. Our results showed that a blood glucose level ≥ 140 mg/dl at admission was associated with a significantly increased risk of mortality from COVID-19. Multivariate logistic regression analysis demonstrated that higher baseline HbA1c was significantly associated with increased mortality. In a study published by Liu et al., higher fasting blood glucose concentration at admission significantly predicted worse outcomes in patients with COVID-19, irrespective of steroid use [13]. In a meta-analysis by Kumar et al. including 16003 patients, the presence of diabetes in COVID-19 patients was associated with a higher mortality rate compared to patients without diabetes [14]. Another observational study from China showed that the presence or absence of diabetes did not affect the outcome in COVID-19 patients [15]. Similarly, the current study did not demonstrate a significant association between preexisting diabetes and increased mortality.

Data regarding the association of COVID-19 and mucormycosis is emerging and is primarily available as case reports. We report four cases of mucormycosis in COVID-19, and all patients had underlying uncontrolled diabetes. All patients received high-dose immunosuppressive agents (tocilizumab or pulse steroids) for management of COVID-19-related cytokine storm, and mortality was 100% in these patients despite aggressive treatment. We believe that profound immunosuppression due to liberal use of immunosuppressive drugs, COVID-19 induced immune dysregulation and uncontrolled diabetes predisposed these patients to mucormycosis. Due to the retrospective nature of this study, the specific predisposing factor cannot be determined. Hanley et al. reported a case of disseminated mucormycosis in a young male with SARS-CoV-2 infection, which was diagnosed postmortem [16]. India contributes to around 40% of the global burden of mucormycosis with an estimated prevalence of 140 cases per million population [17]. The presence of ocular or facial nerve paralysis, orbital involvement, and necrotic lesion in a COVID-19 patient are the red flags that should prompt immediate evaluation for rhino-orbital mucormycosis. Clinicians should be aware of this rare association seen predominantly in the post-COVID-19 period requiring a high index of suspicion to diagnose mucormycosis in post-COVID-19 patients. Delay in diagnosis and treatment of mucormycosis can lead to devastating consequences.

Hypocalcemia is commonly encountered in critically ill patients. The postulated mechanisms responsible for hypocalcemia in these patients include vitamin D deficiency, vitamin D resistance, acquired relative hyperparathyroidism, and decreased 1-alpha-hydroxylase enzyme activity [18]. Increased levels of proinflammatory cytokines like IL-1 beta and IL-6 are primarily implicated in the development of acquired relative hyperparathyroidism by upregulating calcium-sensing receptors on parathyroid cells [19,20]. Hypocalcemia with hyperphosphatemia along with normal renal functions was observed in 3.9% of patients. Serum PTH levels were inappropriately low in the setting of hypocalcemia. We believe that acquired relative hypoparathyroidism could be responsible for this biochemical picture in the affected patients. Although hypocalcemia prevalence increased with increasing severity, this relationship did not reach statistical significance. Hypocalcemia was detected in 44.4% of patients with moderate disease. Another study from North India reported low calcium levels in patients with non-severe COVID-19 compared to healthy controls [21]. These findings suggest that hypocalcemia is prevalent among patients with COVID-19, irrespective of disease severity. The possibility of a direct effect of SARS-CoV-2 effect on the calcium-vitamin D-parathyroid hormone axis cannot be ruled out, requiring large-scale prospective studies to clarify the mechanisms responsible for hypocalcemia in COVID-19. Our findings indicated that low vitamin D level was independently associated with severe disease and increased mortality in ICU patients. In a systematic review by Pereira et al., a positive association was observed between low vitamin D levels and COVID-19 severity [22].

This study highlights the importance of various endocrine manifestations like uncontrolled diabetes, low free T3, and low vitamin D3, which have an impact on the outcome of patients with COVID-19. We reported the association of new-onset thyrotoxicosis due to subacute thyroiditis with severe COVID-19. The current study design excluded patients who received corticosteroids before thyroid function tests, reflecting the accurate picture of thyroid function abnormalities encountered in COVID-19 patients.

Limitations

There are still some limitations in our study. First, this was a retrospective study; therefore, a causal relationship could not be established. Second, PTH concentration was not routinely measured in all patients with hypocalcemia. Third, baseline cortisol levels were not measured in most patients, making it difficult to assess the relationship between cortisol levels and outcomes. Fourth, this was not a true case-control study, so the strength of association might not be the true representative of actual risk.

## Conclusions

During the ongoing pandemic, endocrine manifestations of COVID-19 remain largely unexplored. A high index of suspicion is necessary to timely diagnose post-COVID-19 mucormycosis, especially in patients with underlying diabetes mellitus. New-onset thyrotoxicosis in COVID-19 patients is largely due to subacute thyroiditis and does not affect the outcome. Hypocalcemia is also commonly encountered in patients with moderate disease and those with critical COVID-19 and relative hypoparathyroidism could account for a few cases. Clinicians must be aware of these possibilities in routine practice, especially while managing COVID-19 survivors.
